# Learning the Hidden Signature of Fetal Arch Anatomy: a Three-Dimensional Shape Analysis in Suspected Coarctation of the Aorta

**DOI:** 10.1007/s12265-022-10335-9

**Published:** 2022-10-27

**Authors:** Uxio Hermida, Milou P. M. van Poppel, David F. A. Lloyd, Johannes K. Steinweg, Trisha V. Vigneswaran, John M. Simpson, Reza Razavi, Adelaide De Vecchi, Kuberan Pushparajah, Pablo Lamata

**Affiliations:** 1 Department of Biomedical Engineering, School of Biomedical Engineering and Imaging Sciences, King’s College London, St Thomas’ Hospital, 5Th Floor Becket House, 1 Lambeth Palace Road, London, SE1 7EH UK; 2 Department of Cardiovascular Imaging, School of Biomedical Engineering and Imaging Sciences, King’s College London, St Thomas’ Hospital, London, SE1 7EH UK; 3 Department of Perinatal Imaging, School of Biomedical Engineering and Imaging Sciences, King’s College London, St Thomas’ Hospital, London, SE1 7EH UK; 4 Department of Congenital Heart Disease, Evelina London Children’s Hospital, London, SE1 7EH UK; 5 Harris Birthright Centre, Fetal Medicine Research Institute, King’s College Hospital, London, UK

**Keywords:** Clinical Biomarker, Computational Anatomy, Congenital Heart Disease, Machine Learning, Magnetic Resonance Imaging, Statistical Shape Modeling

## Abstract

Neonatal coarctation of the aorta (CoA) is a common congenital heart defect. Its antenatal diagnosis remains challenging, and its pathophysiology is poorly understood. We present a novel statistical shape modeling (SSM) pipeline to study the role and predictive value of arch shape in CoA in utero. Cardiac magnetic resonance imaging (CMR) data of 112 fetuses with suspected CoA was acquired and motion-corrected to three-dimensional volumes. Centerlines from fetal arches were extracted and used to build a statistical shape model capturing relevant anatomical variations. A linear discriminant analysis was used to find the optimal axis between CoA and false positive cases. The CoA shape risk score classified cases with an area under the curve of 0.907. We demonstrate the feasibility of applying a SSM pipeline to three-dimensional fetal CMR data while providing novel insights into the anatomical determinants of CoA and the relevance of in utero arch anatomy for antenatal diagnosis of CoA.

**Graphical abstract:**

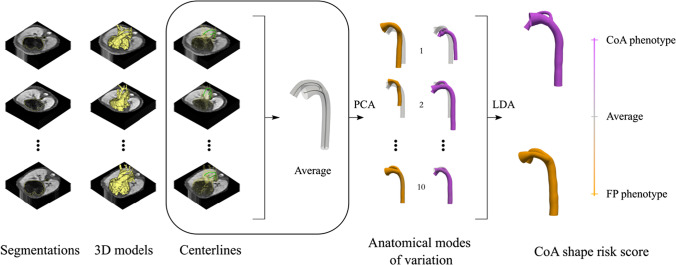

**Supplementary Information:**

The online version contains supplementary material available at 10.1007/s12265-022-10335-9.

## Introduction

Coarctation of the aorta (CoA) is one of the most common congenital heart defects, affecting 7–8% of all live births with congenital heart disease [1] and is characterized by a discrete narrowing in the region of the aortic isthmus after postnatal closure of the arterial duct (AD) with a variable degree of hypoplasia of the aortic arch. Prenatal diagnosis of neonatal or duct-dependent CoA is associated with reduced morbidity and mortality [2] but remains challenging, with false positive rates of diagnosis ranging from 20–30% up to 80% [3–5], posing unnecessary stress on parents and a burden on healthcare systems because, after birth, these babies need to be observed in a hospital setting to check for clinical and echocardiographic signs of aortic arch obstruction [6–8].

Standard, two-dimensional (2D) fetal ultrasound imaging planes include visualization of the transverse and distal aortic arch in axial views. In this projection, the aortic isthmus may be out of plane and difficult to image. Sagittal views of the aortic arch depend on the fetal lie and cannot be consistently obtained. Various ultrasound metrics have been explored and reported aiming to improve antenatal diagnosis of CoA [4, 9–13]. However, such metrics are reported to have relatively low specificity, as well as poor generalizability [4]. Other studies have focused on improving our understanding of the pathophysiological mechanisms leading to neonatal CoA [14–18], but no definitive cause for the disease has been identified to date. In a recent observational study, the utility of novel, high-resolution, three-dimensional (3D) fetal cardiac magnetic resonance imaging (CMR) for antenatal diagnosis of CoA was investigated, and novel anatomic and flow-related biomarkers predictive of CoA were described [19]. This preliminary study demonstrated an important role for the spatial relationship between the aorta and AD in the prediction of CoA. However, as in previous studies, the role of shape in CoA was assessed using observer-dependent 2D measurements, not fully capturing the complexity of 3D arch geometry.

Digital twin technologies have emerged as a supporting tool to inform clinical decision-making and improve understanding of pathophysiological mechanisms [20]. Among those, statistical shape modeling (SSM) has been used to study the relationship between cardiovascular conditions and risk factors [21], characterize cardiac remodeling [22], or improve understanding of pathophysiological mechanisms [23]. In adults with repaired CoA, SSM has been used to understand the interplay between shape and function [24, 25]. However, SSM has not been applied to fetal cardiac imaging due to the lack of 3D data.

Recent developments in fetal CMR now allow reliable motion-correction of the fetal thorax [26], resulting in detailed 3D cardiac volumes. This creates an opportunity to apply SSM techniques and fully explore the role of arch geometry in duct-dependent CoA before birth. In this study, we aim to:

- Develop and apply a SSM pipeline to novel 3D fetal CMR data to gain more insights into the role of fetal arch shape in cases with suspected neonatal CoA.
- Quantify the potential of detailed fetal arch shape analysis to differentiate between FP and CoA cases using 3D fetal CMR.

## Methods

### Study Population and Data Acquisition

Between July 2015 and April 2021, women carrying a fetus with suspected CoA were offered fetal CMR at discretion of their fetal cardiologist after one or more (clinical) fetal echocardiography examinations. General fetal CMR exclusion criteria included maternal weight > 125 kg, severe claustrophobia, inability to give informed consent, or < 18 years of age. Fetuses with external compression of left heart structures (i.e., severe congenital diaphragmatic hernia) or abnormal ventriculoarterial connections (i.e., transposition of the great arteries, double outlet right ventricle) were excluded from analysis.

Fetal CMR examinations consisted of multiple T2-weighted “black-blood” standard single-shot fast spin echo sequences covering the fetal thorax acquired on a 1.5 Tesla Ingenia MR system (Philips, Best, the Netherlands), with repetition time (TR), 20,000 ms; echo time (TE), 80 ms; flip angle, 90°; voxel size, 1.25 × 1.25 mm; slice thickness, 2.5 mm; sensitivity encoding (SENSE) factor, 2; partial Fourier factor, 0.547; and slice duration, 546 ms. No external gating, fetal or maternal sedation, or intravenous contrast were used.

**Fig. 1:**
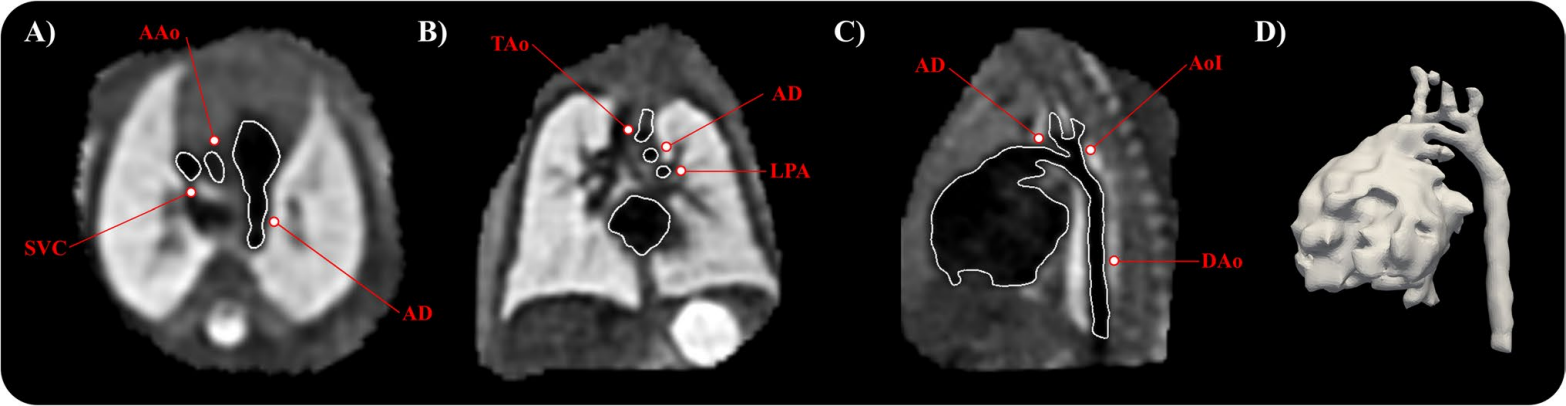
“Black-blood” motion-corrected 3D CMR of the fetal thorax and corresponding segmentation. **A** Transverse view at level of three vessel view. **B** Coronal view showing transverse aortic and ductal arch. **C** Sagittal view showing aortic isthmus and arterial duct (AD). **D** Corresponding three-dimensional mesh. Relevant anatomical locations are highlighted in red. AAo, ascending aorta; AoI, aortic isthmus; DAo, descending aorta; LPA, left pulmonary artery; TAo, transverse aortic arch

The 2D data was processed contemporaneously using a clinically validated motion-corrected slice-volume registration method [26], producing high-resolution 3D volumes (range 0.55–0.75 mm isotropic). Segmentation of the fetal heart was conducted using a semi-automatic thresholding tool from ITK-SNAP (version 3.6.0) [27] with manual refinement of region of interest for clinical reporting purposes at time of scan by a clinician with fetal CMR experience (M.P.M.v.P., D.F.A.L., J.K.S.). Note that different intensity thresholds were used for each case, as the contrast of reconstructions is case-dependent. All reconstructions and segmentations acquired with research consent were collected and inspected for adequate delineation of vascular structures before inclusion. Where required, reconstructions were repeated. Segmentations of the main cardiovascular structures (ascending aorta (AAo), aortic arch, AD, main pulmonary artery (MPA), and branch pulmonary arteries) were refined manually to allow adequate centerline extraction, conducted by a clinician (M.P.M.v.P.) with 5 years of fetal CMR experience. Images were reoriented according to fetal left-to-right and inferior-to-superior direction (see Fig. 1). Subsequently, 3D models were generated using a discrete Marching Cubes algorithm and smoothed with a windowed sinc function.

Cardiac outcomes were collected using hospital records. True positive CoA was defined as duct-dependent CoA requiring surgical repair within 28 days of age. False positive (FP) was defined as discharged from hospital without surgical intervention after closure of the AD. At our institution, follow-up of FP cases up to 1 year of age is standard policy to detect late development of CoA. No healthy control fetuses were available for study.

### Statistical Shape Modeling: Pipeline

Motion-corrected 3D fetal CMR images and their corresponding segmentations, although validated and robust [19, 26], are prone to local surface irregularities. Recent developments in 3D fetal CMR offer new options for non-rigid motion correction to reconstruction quality [28]. However, this was not applied to the present retrospective cohort. Therefore, to build a robust statistical shape model from fetal CMR data, we opted for a centerline-based parametric SSM approach similar to the one described in [29]. A three-step semi-automatic pipeline was designed. All functionalities were implemented using open-source Python toolkits, such as the Visualization Toolkit (VTK) [30] or the Vascular Modeling Toolkit (VMTK) [31].

#### Landmark Protocol and Centerline Extraction

**Fig. 2:**
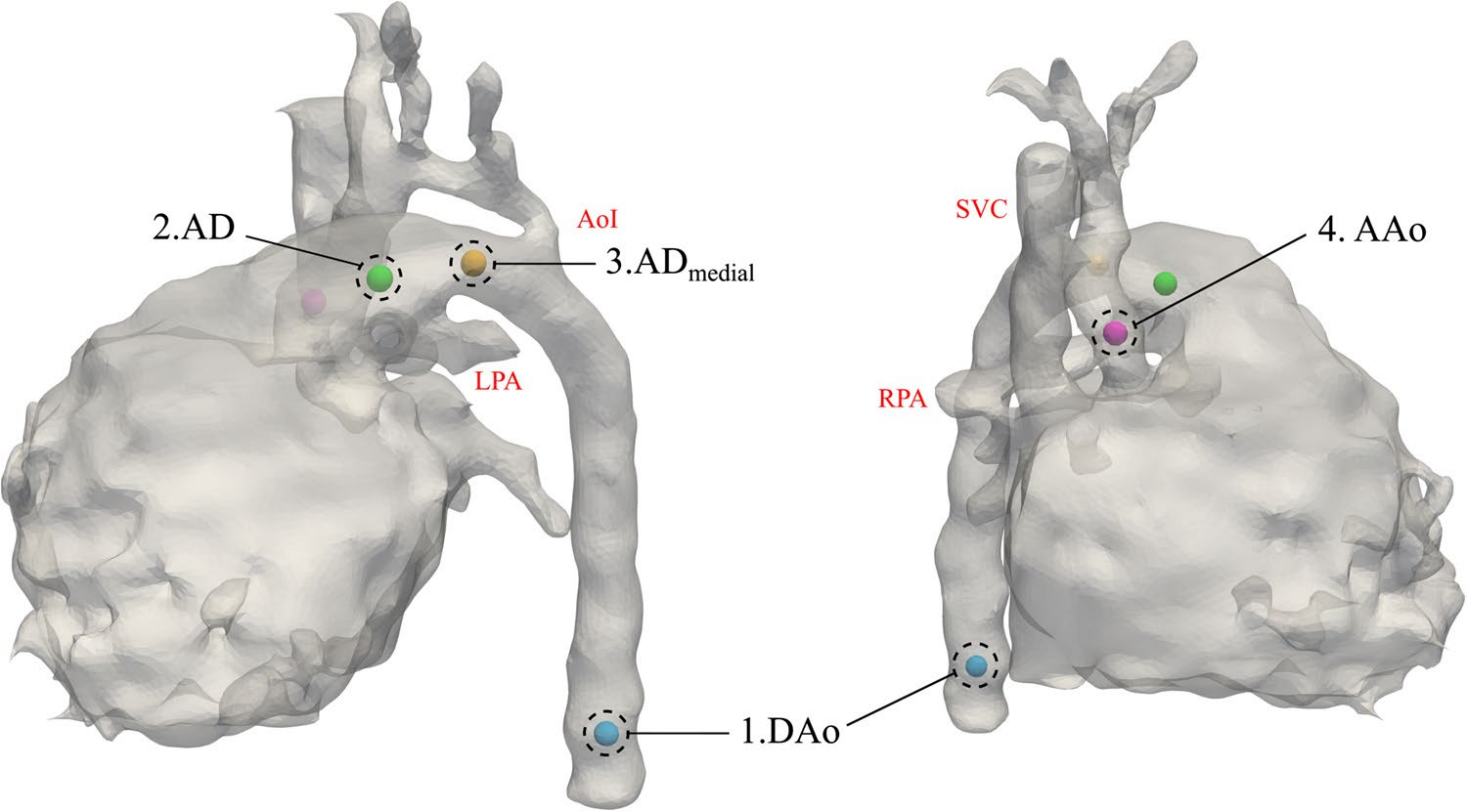
Anatomical landmarks needed to extract the centerlines. (1) Descending aorta (DAo) at level of the diaphragm; (2) arterial duct (AD) at pulmonary artery bifurcation; (3) medial point of AD; (4) ascending aorta (AAo) at the level of RPA. AoI, aortic isthmus; LPA, left pulmonary artery; RPA, right pulmonary artery; SVC, superior vena cava

Three main arterial segments are involved in the development of neonatal CoA: AAo to aortic isthmus (defined as the segment distal to the origin of the left subclavian artery and proximal to insertion of the AD), AD, and descending (thoracic) aorta (DAo). T2-weighted “black-blood” reconstructions of the fetal thorax allow a detailed visualization of all those segments due to the contrast with the fluid-filled lungs. However, they do not allow detailed visualization of intracardiac structures (e.g., heart valves). In addition, the AAo at valvular level can be difficult to delineate due to the position central in the beating fetal heart, especially in cases with underdeveloped left heart structures. Therefore, to ensure robust centerlines with proper correspondence between cases, we opted for a semi-automatic centerline extraction approach that relies on 4 anatomical landmarks (see Fig. 2): (1) DAo at level of the diaphragm; (2) AD at pulmonary artery bifurcation; (3) medial point of AD; and (4) AAo at the level of right pulmonary artery (RPA).

Due to the connection between the AAo and AD through the heart, to ensure a proper centerline extraction, centerlines were extracted in two steps. First, using landmarks 1 and 2, the centerline from the DAo until the pulmonary artery bifurcation was extracted. Then, as a safety measure, the medial section of the AD was clipped from the 3D model using landmark 3. Using the clipped model, together with landmarks 1 and 4, the centerline from the DAo until the AAo was computed. Centerlines were then used to geometrically decompose the bifurcating anatomy. As a result, the final step is to merge the two extracted centerlines at the objectively defined bifurcation origin. For a more detailed description of the decomposition of arterial bifurcations, the reader is referred to [31, 32]. Due to length differences between segments, centerlines were independently resampled to a user-defined (evenly spaced) set of points: 25 points for AAo and AD and 51 points for DAo.

#### Centerline Alignment

**Fig. 3:**
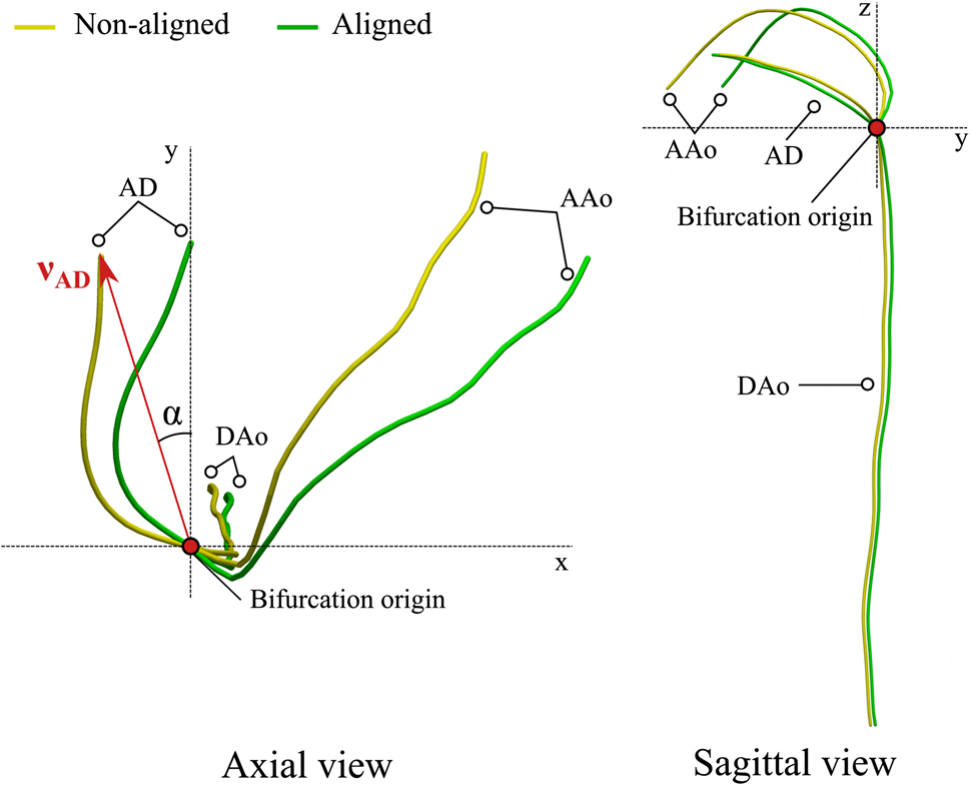
Schematic representation of the alignment of centerlines prior to building the statistical shape model. An example of non-aligned centerlines is shown in yellow; the aligned in green. Using the bifurcation origin and a vector *v*_AD_ from the bifurcation origin until the start of the arterial duct (AD), the non-aligned centerline is rotated with respect to the vertical axis *Z* using the angle *α*. DAo, descending aorta; AAo, ascending aorta

Alignment of shapes is a key step in the statistical shape modeling pipeline. First, images were aligned with respect to the fetal anatomical left-to-right and inferior-to-superior directions by a clinician (M.P.M.v.P) with 5 years of fetal CMR experience as part of the clinical reporting service. Then, using the aligned images, centerlines were extracted. After extraction and geometrical decomposition, all centerlines were spatially aligned with respect to their bifurcation origin. Next, to compensate for potential big differences in the direction of the AD and AAo, the AD direction (a vector VAD from the bifurcation origin until the start of the arterial duct (AD)) was used to rotate the centerlines with respect to the vertical axis Z (see Fig. 3). No scaling was performed to maintain size information, as the relative size of each segment is known to be highly predictive of CoA and related to its pathophysiology.

#### Building the Statistical Shape Model

After extraction of centerlines, information of shape is encoded by the coordinates for each point and their associated maximal inscribed radius. A total of *N* = 101 points were used to construct a feature vector of length 4 N for each case. A principal component analysis (PCA) can then be used to reduce the dimensionality of the 404 degrees of freedom and build the statistical shape model.

PCA finds the directions that maximize the variance in the observed shapes around the population average, capturing the most common changes in a cohort (i.e., the linear directions of anatomical change from the population average, commonly referred as anatomical modes of variation or PCA modes). PCA modes are ranked by the amount of variance explained, so that first modes represent the most robust shape features to acquisition and segmentation noise. As a result, the parametric space is reduced, and each case can be described by a small set of shape coefficients that defined the linear combination of the first k PCA modes. A two-tailed Student’s *t*-test was used to determine differences between shape coefficients for each subgroup in the population (CoA/FP). *P*-values < 0.01 were considered statistically significant. 3D extreme shapes (i.e., ± 3SD from the average shape) for each PCA mode were reconstructed. For more details about how the statistical shape model is built as well as a step-by-step description of the 3D reconstruction of extreme shapes for each axis, the reader is referred to the Supplementary Material.

### Finding the Signature Shape of CoA

#### Linear Discriminant Analysis

After PCA, shape information is decomposed into several PCA modes. Therefore, a Fisher linear discriminant analysis (LDA) was used to find the linear combination of PCA modes that best discriminates between FP and CoA cases, thus obtaining a single anatomical deformation axis (LDA mode) [33]. The resulting LDA mode depends on the PCA modes included. Therefore, 25 different LDA models were built with an increasing number of PCA modes. The area under the receiver operating characteristic (AUC) in resubstitution (RS) and leave-one-out cross-validation (LOOCV) was explored to find the optimal number of PCA modes that minimizes the risk of overfitting while capturing as much shape variability as possible and maximizing classification performance. Each vascular fetal shape can be then characterized by a single score along the optimal LDA axis, referred as CoA shape risk score (i.e., Z-score), and extreme phenotypes can be derived to get qualitative pathophysiological insights.

Finally, the discriminatory performance of the CoA shape risk score was compared against simple geometrical descriptors. The coefficient of determination (*R*^2^) between the CoA shape risk score and each descriptor was also studied and used to get better insights into the features captured by the CoA shape risk score. All metrics were automatically extracted using the patient-specific centerlines and their captured surface. For more details, including a visual description of the geometrical descriptors, the reader is referred to the Supplementary Material.

#### Robustness of Findings

The robustness of the qualitative (i.e., optimal LDA shape phenotypes) and quantitative (i.e., AUC) findings was explored to ensure capturing the right pathophysiological signature. First, 5 different SSM were built using random subsets of the population of increasing size: 50%, 60%, 70%, 80%, and 90%. The convergence of the resulting extreme phenotypes and the discriminatory performance of each of those axes in RS and LOOCV were assessed. A stratified random sampling approach was chosen to select the samples for each model, thus keeping constant the proportion of FP and CoA cases. Moreover, each model was run 10 times with different randomly selected subsets to have a better estimate of their performance with independence of the cases selected.

Each anatomy can be reconstructed by sequentially adding the information contained in each subsequent PCA mode to the average shape. Therefore, we explored the reconstruction error (i.e., root mean squared error (RMSE)) between each reconstructed shape depending on the PCA modes included and the reference shape for each case. RMSE was explored for each centerline segment separately (i.e., AAo, AD, DAo), for all three grouped segments, and the centerline points closer to the isthmal insertion (i.e., half of the points for each centerline segment).

## Results

### Study Population Characteristics

In total 112 patients (median gestational age 32 weeks, range 29–38) were included for statistical shape analysis (i.e., PCA). No cases were excluded based on image quality. Outcome data was available for 108/112 patients, and 43/108 (40%) were agreed for surgical repair of CoA in the neonatal period. One fetus in the FP group developed CoA after this time period and had surgical repair at 2.9 months. Median follow-up of FP cases was 13.6 months (range 1.7–62.6 months), and 17/64 (27%) patients had been discharged from cardiac follow-up at time of writing. Postnatally confirmed associated cardiac lesions are depicted in Table 1.

**Table 1:** Study population characteristics: postnatally confirmed associated cardiac lesions

	CoA (*n* = 43)	FP (*n* = 65)
Gestational age at fetal CMR (weeks)	32.1 (1.7)	32.2 (1.7)
Atrioventricular septal defect	7 (16)	0
Atrial septal defect	1 (2)	3 (5)
Ventricular septal defect	18 (42)	17 (26)
Aberrant right subclavian artery	0	5 (8)
Persistent left superior vena cava	4 (9)	16 (25)
Partial anomalous venous drainage	0	7 (11)
Bicuspid aortic valve	23 (54)	8 (12)
Mitral valve anomalies	6 (14)	4 (6)
Tricuspid valve anomalies	2 (5)	0
Pulmonary valve stenosis	0	1 (2)
Dextrocardia	1 (2)	0
Interrupted inferior vena cava	1^∗^ (2)	3^§^ (5)

Data are presented as mean (SD) or *n* (%). *CoA* coarctation of the aorta, *FP* false positive

^*^Isolated finding

^§^Left atrial isomerism

### Shape Changes in CoA

PCA resulted in the first 10 anatomical modes of variation capturing 86% of shape variation in the population. FP and CoA cases differed significantly in modes 1, 2, and 4 (*P*-value < 0.01). For a detailed visual representation of the three significant PCA modes, see Supplementary Videos 1–3. PCA modes with larger differences between the two subgroups showed that FP cases tend to have a more aneurysmal AD inserting laterally into the aortic arch and DAo while confirmed CoA cases tend to have a more proximal insertion of the isthmus into the superior aspect of a non-aneurysmal AD with increased aortic isthmus angulation. This feature is reflected as an almost perpendicular angle between the AD and aortic isthmus and the vertical co-planarity between the AD, aortic isthmus, and DAo insertion points. Global size changes, including the absolute and relative length of each segment, were also captured by several modes. Changes in cross-sectional diameter were also captured across all 10 modes, showing that CoA cases tend to have a smaller AAo and aortic isthmus to AD ratio. It is noteworthy that, even if visual inspection of the PCA modes of variation allows their interpretation, PCA modes capture these changes and also identify other subtle remodeling patterns.

### The Signature Shape of CoA

#### Optimal Discriminant Axis of Anatomical Variation

**Fig. 4:**
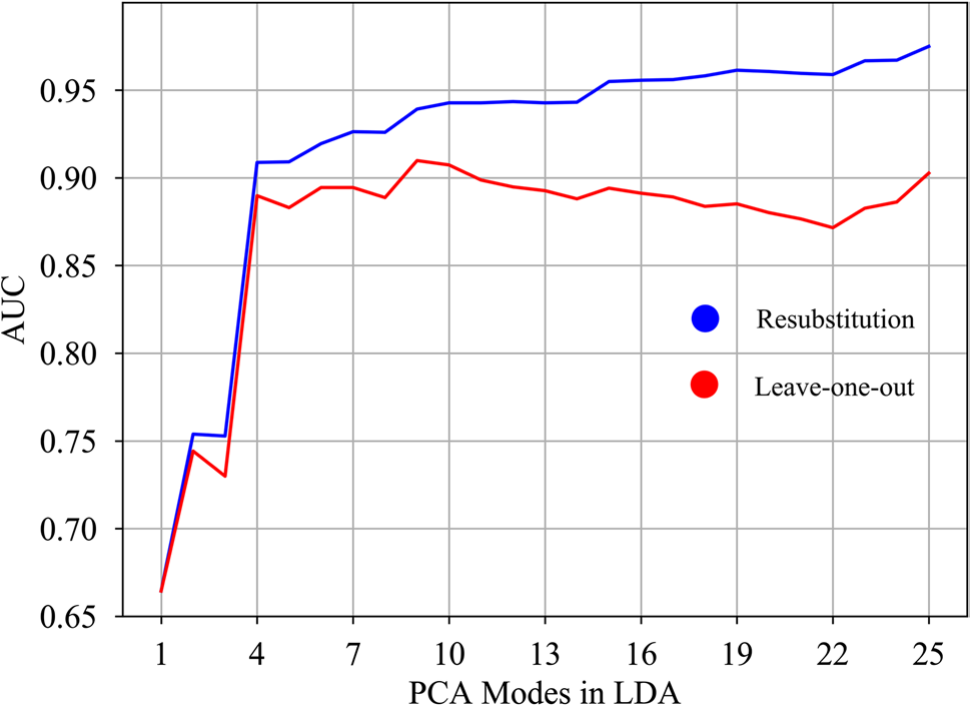
Area under the curve in resubstitution (RS) and leave-one-out cross-validation (LOOCV) depending on the number of PCA modes included in the optimal LDA model

**Fig. 5:**
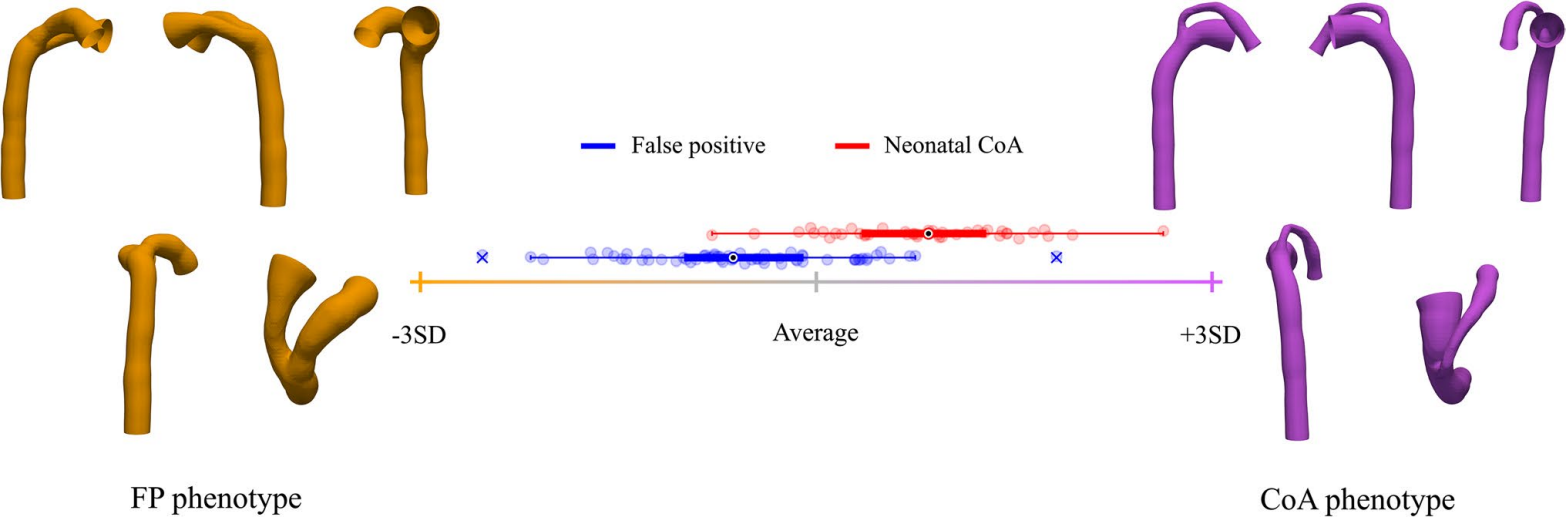
Optimal LDA between neonatal coarctation of the aorta (CoA) and false positive (FP) cases. Box-plots represent the CoA (red) and FP (blue) CoA shape risk score distributions along the axis. Orange cross and shape show -3SD from the average population shape (gray; CoA risk score 0); purple cross and shape, + 3SD. For more details, see Supplementary Video 4

Excellent AUC in RS and LOOCV was obtained as a combination of the first 4 PCA modes (see Fig. 4). However, the addition of up to 10 PCA modes refined the obtained LDA axis, increasing the AUC in RS without overfitting the data. After mode 10, the LDA model started overfitting (see gap between AUC in RS and LOOCV in Fig. 4). Therefore, the linear combination of the first 10 modes of anatomical variation was considered as the optimal LDA mode. The resulting CoA shape risk score (i.e., Z-score) was able to classify FP and CoA cases with an AUC of 0.943 in RS and 0.907 in LOOCV. LDA extreme shapes (see Fig. 5 and Supplementary Video 4) show the aforementioned shape changes between the 2 groups, and, in addition to expected great artery asymmetry, variation in aortic isthmus insertion and course of the AD (aneurysmal in FP) as well as aortic arch (superior to AD in CoA).

The CoA shape risk score outperformed all simple geometrical descriptors. Best prediction was obtained with the projected vertical angle between AD and aortic isthmus (Ang_vert_- AUC 0.809) and cross-sectional diameter of the distal AAo (Diam_dAAo_—AUC 0.762). Larger *R*^2^ values were obtained for Ang_vert_, Diam_dAAo_, and vertical displacement of the transverse aortic arch with respect to the medial AD (Disp:Vert). For more details about the individual performance of each descriptor and *R*^2^ values, see Table S1 in the Supplementary Material.

#### Robustness of Findings

**Fig. 6:**
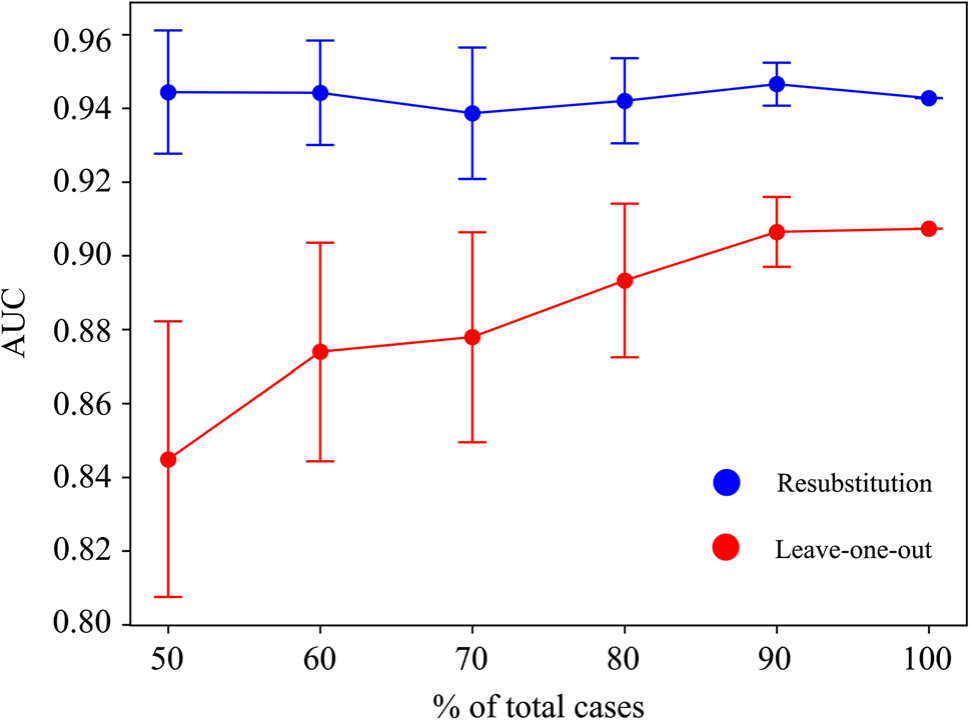
Error bars showing the mean ± SD of the area under the curve (AUC) depending on the number of cases used in the LDA

**Fig. 7:**
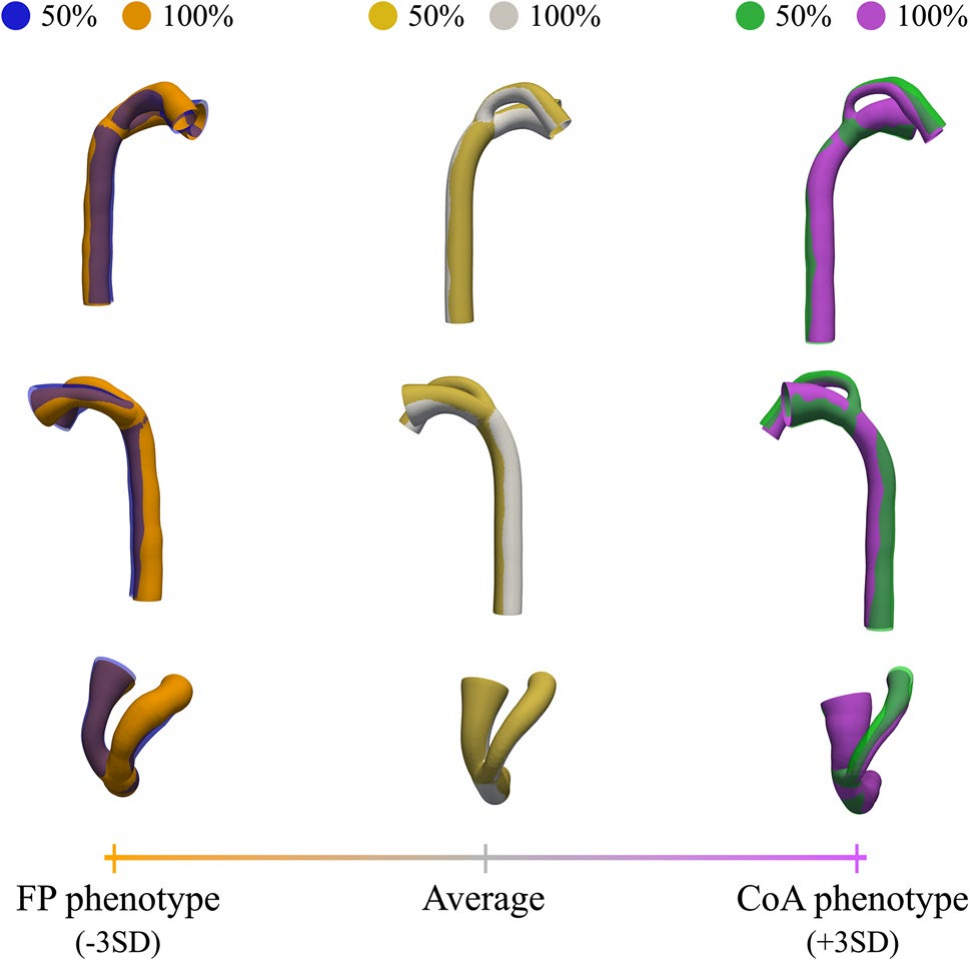
Optimal LDA using 50% or 100% of the population. Each shape represents the mean of all shapes from the 10 different LDA models built with 10 random subsets of the population. Orange cross and shape (FP phenotype) show -3SD from the average shape of the 100% population (shown in gray); purple cross and shape (CoA phenotype), + 3SD. The 50% population shapes are overlaid: blue shape shows -3SD from the average shape of the 50% population (yellow); green shape, + 3SD. See Supplementary Videos 5–7 for more details

The discriminatory performance of the optimal LDA mode depending on the number of cases used, both in RS and LOOCV, is shown in Fig. 6. The more cases used to build the SSM, the more the convergence towards the performance of the 100% SSM. The gap between RS and LOOCV (i.e., overfitting) decreased with the number of cases used. The fewer cases used, the more variance in the LOOCV performance, which is a consequence of the subset of cases selected to build the model. However, such performance differences were not reflected as significant changes in the resulting phenotypes. Figure 7 shows the change in the LDA phenotypes when using 50% or 100% of the population. Each shape represents the mean of all shapes resulting from the 10 different LDA models built with 10 random subsets of the population. Most differences were observed in the CoA extreme shape, mostly in the AAo segment. For a detailed visualization of each phenotype using each percentage of the population (i.e., 50%, 60%, 70%, 80%, 90%, 100%), see Supplementary Videos 5–7.

Reconstruction of individual cases with a progressive number of PCA modes (see Fig S4) showed that 10 PCA modes allowed a good anatomical reconstruction (RMSE: 0.17 mm), particularly around the bifurcation area (RMSE: 0.07 mm). Accurate reconstruction of the DAo was achieved with 7 PCA modes, while the RMSE for the AAo and AD slightly decreased up to PCA mode 15.

## Discussion

In this study, we developed and applied a novel SSM pipeline to 3D fetal CMR data. In addition, we have thoroughly explored the role of shape in suspected CoA in utero and potential morphological discriminants between CoA and FP cases. We have made a virtual cohort of 1000 synthetic cases available for the community [https://doi.org/10.5281/zenodo.6256918]. Data includes centerlines, captured 3D surfaces, and shape coefficients, corresponding CoA shape risk scores.

### The Interplay Between Shape and Blood Flow

Our results demonstrate that fetuses with postnatally confirmed CoA have a more superior course of the aortic arch with an almost vertical insertion of the aortic isthmus into a non-tortuous AD with distinct proximal displacement, while FP cases show a more aneurysmal AD with lateral insertion into the aortic isthmus and descending aorta. In the preliminary CMR study from our institution [19], we observed that aortic and isthmal flows appear to vary closely with the angulation and displacement of the aortic isthmus. From a mechanistic point of view, the observed shape features and extreme phenotypes in our study agree with this study and theories proposed over 50 years ago [15, 16] and support the theory that the aortic arch becomes a functional branch of the AD. We show that in extreme FP, there is a direct pathway from the aorta towards the DAo. Conversely, in extreme CoA cases, the AD has a direct course to the DAo. We hypothesize that variations in shape and potential abnormal blood flow patterns could affect the arterial wall (e.g., wall shear stress) and vascular remodeling. Computational methods, such as the one presented, can provide new mechanistic insights into the interplay between shape and blood flow. Further studies are required to elucidate the association between vascular shape and underlying flow in the aortic and ductal arch, in addition to other factors such as genetics, underlying vasculopathy, migration of ductal tissue, and cerebroplacental changes.

### Robustness of Findings

We explored how sample size would affect the pathophysiological insights derived from the analysis. Sample size affected the discriminant performance of the optimal LDA mode in LOOCV showing overfitting to the data and should be considered for its clinical utility (see Fig. 6). However, no significant changes in the resulting LDA phenotypes were seen (see Fig. 7 and Supplementary Videos 5–7). These robust shape patterns—independent of the number of included cases—reinforce the confidence in the pathophysiological insights derived from the model. Moreover, reconstruction error showed good agreement between reconstructed shapes and reference anatomies using 10 PCA modes. Finally, the uniqueness of the axis of remodeling to the choice of the experimental group (i.e., including control cases) has been demonstrated elsewhere [34], which is an additional evidence of the nominal impact of potential sources of variability in our pipeline (number of cases included, manual segmentations, and landmarks).

### Predictive Value of Shape in CoA

Overall, shape features resulting from the SSM agree with previous studies. Commonly described parameters such as differences in cross-sectional diameter and Isthmus:Duct ratio were captured by the SSM. The parameter as described in the preliminary CMR study [19] reflecting the “posterior shelf” (Disp:DAo) was captured by several modes as well as the discriminant axis (see Supplementary Videos 1–4). However, note that there is overlap in patient groups between both studies. Aortic arch angle measurements using fetal echocardiography have been proposed to improve accurate prenatal identification of neonatal CoA [13, 35]. We did not reproduce these measurements due to inherent differences between the used imaging methods; however, aortic arch angular changes were captured by the statistical shape model.

We attempted to automate the extraction of simple geometrical metrics from 3D fetal CMR images with the aim of reducing inter- and intra-observer variability and extraction time. None of the metrics outperformed our CoA shape risk score. Many of the explored morphological determinants, as well as those suggested in the literature, were captured by the statistical shape model. The improved performance of the CoA shape risk score might indicate that it is the combination of several anatomical features that best discriminates between FP and CoA cases. Interestingly, the Angvert obtained a relatively high AUC compared to previous work [19] likely due to its automatic extraction using a robust geometrical decomposition of the bifurcation [32]. The objective decomposition of arterial segments is not trivial, considering the complex spatial relationship of the arches, and, therefore, manual measurements from 2D images can be prone to high inter-observer variability and lack of robustness, which could explain differences in performance across different cohorts [4]. Despite the excellent discriminative performance of the CoA shape risk score, there is still an overlap of FP and CoA scores around the average. This suggests that anatomical biomarkers of the great arteries alone are not enough and highlights the importance of flow in the pathophysiology of CoA. Thus, there is a need for integration of robust anatomically derived biomarkers with other functional biomarkers to further reduce the overlap between populations and improve prediction of neonatal CoA.

### Future Work

Future studies should focus on the relationship between the CoA shape risk score and other parameters such as blood flow or ventricular changes. We have previously observed that FP fetuses differ from healthy age-matched fetuses [19]. Therefore, arch shape differences should be compared between healthy controls, FP, and neonatal CoA. Advances in fetal CMR [36, 37] could also enable more detailed studies of myocardial and ventricular remodeling in fetuses with suspected CoA. Given the different manual steps along the presented pipeline, future studies should explore automatic approaches for segmentation of fetal cardiac anatomies and the landmarks required for the current pipeline. Additionally, computational numerical methods, such as computational fluid dynamics simulations, could be used to explore the hypothesis of local hemodynamic changes in the region of the aortic isthmus. Finally, several proof-of-concept studies have reported the feasibility of using echocardiography to obtain 3D reconstructions of the fetal circulation [38, 39]. This offers the opportunity to apply the presented methodology to fetal echocardiography.

### Limitations

First, the SSM was constructed using semi-automatic segmentations with manual refinements of fetal CMR. We aimed to reduce the impact of both image and segmentation quality by expert revision of clinical segmentations and by using a centerline-based SSM approach; however, it could still impact results. Fully automatic segmentation tools could reduce manual input and processing time in the future. Secondly, images were aligned with respect to fetal left-to-right and inferior-to-superior directions using a set of user-defined landmarks (prone to inter-observer variability). Similarly, landmarks used to extract centerlines are observer-dependent and can result in length differences for each segment. However, both were performed by one observer to ensure consistency, and the LDA was limited to 10 PCA modes, which should be robust to inconsistencies in the labeling process. Third, radial features captured by the centerlines approximate the minimum vascular diameter and the surfaces captured by each centerline are considered tubular at each section. This might not reflect non-tubular vascular structures. We did not include aortic branches in our current analysis, as their anatomy lacks topological consistency between cases. However, previous reports described poor specificity of distance between aortic branches [5, 19]. Finally, this is a retrospective study, and we did not divide our cohort in subgroups according to comorbidities (i.e., ventricular septal defect, persistent left superior vena cava, aberrant right subclavian artery, bicuspid aortic valve, and partial anomalous pulmonary venous drainage). No external validation cohort was available to test our risk score; therefore, a prospective study should be conducted to evaluate the reliability and clinical utility of our findings. Only third trimester fetuses were included due to limitations inherent to fetal CMR. This could be of relevance to the shape phenotypes observed as for example, an aneurysmal duct is more frequently seen in third trimester fetuses. New developments could allow successful earlier fetal CMR [28] creating opportunities to compare arch morphology across gestation.

## Conclusion

We demonstrated a novel application of statistical shape modeling to 3D fetal CMR data, providing unique opportunities for objective exploration of the complex in utero arch geometry in fetuses with suspected CoA. In addition, SMM-derived shape biomarkers show potential to improve antenatal diagnosis of duct-dependent CoA.

## Supplementary Information

Below is the link to the electronic supplementary material.

Supplementary file1 (PNG 1984 KB)

Supplementary file2 (PNG 1967 KB)

Supplementary file3 (PNG 7253 KB)

Supplementary file4 (PNG 1078 KB)

Supplementary file5 (MP4 9374 KB)

Supplementary file6 (MP4 8966 KB)

Supplementary file7 (MP4 9433 KB)

Supplementary file8 (MP4 7709 KB)

Supplementary file9 (MP4 367 KB)

Supplementary file10 (MP4 285 KB)

Supplementary file11 (MP4 323 KB)

Supplementary file12 (PDF 13.1 MB)

## Abbreviations

AAo: Ascending aorta
AD: Arterial duct
AUC: Area under the receiver operating characteristic
CoA: Coarctation of the aorta
DAo: Descending aorta
CMR: Cardiac magnetic resonance imaging
FP: False positive
LDA: Fisher linear discriminant analysis
LOOCV: Leave-one-out cross-validation
MPA: Main pulmonary artery
PCA: Principal component analysis
RMSE: Root mean squared error
RPA: Right pulmonary artery
RS: Resubstitution
SSM: Statistical shape modeling
2D: Two-dimensional
3D: Three-dimensional
