# Supplementary figures and images for: Learning the Hidden Signature of Fetal Arch Anatomy: a Three-Dimensional Shape Analysis in Suspected Coarctation of the Aorta
Source: J Cardiovasc Transl Res. 2022 Oct 27;16(3):738–47. doi: 10.1007/s12265-022-10335-9 (PMC10299929; doi:10.1007/s12265-022-10335-9)

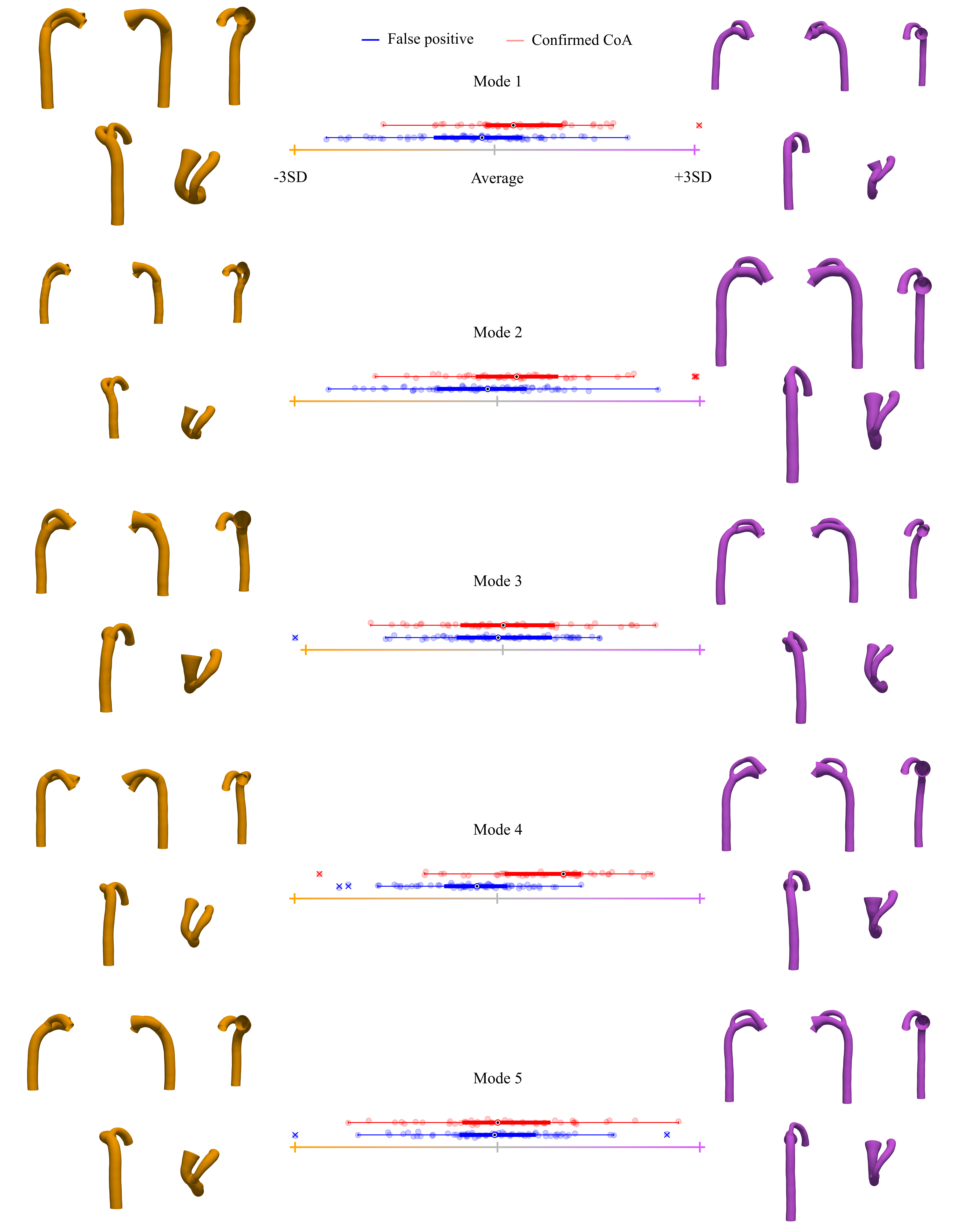

Supplement: Supplementary file 1 — (PNG 1984 KB) [file 12265_2022_10335_MOESM1_ESM.png]

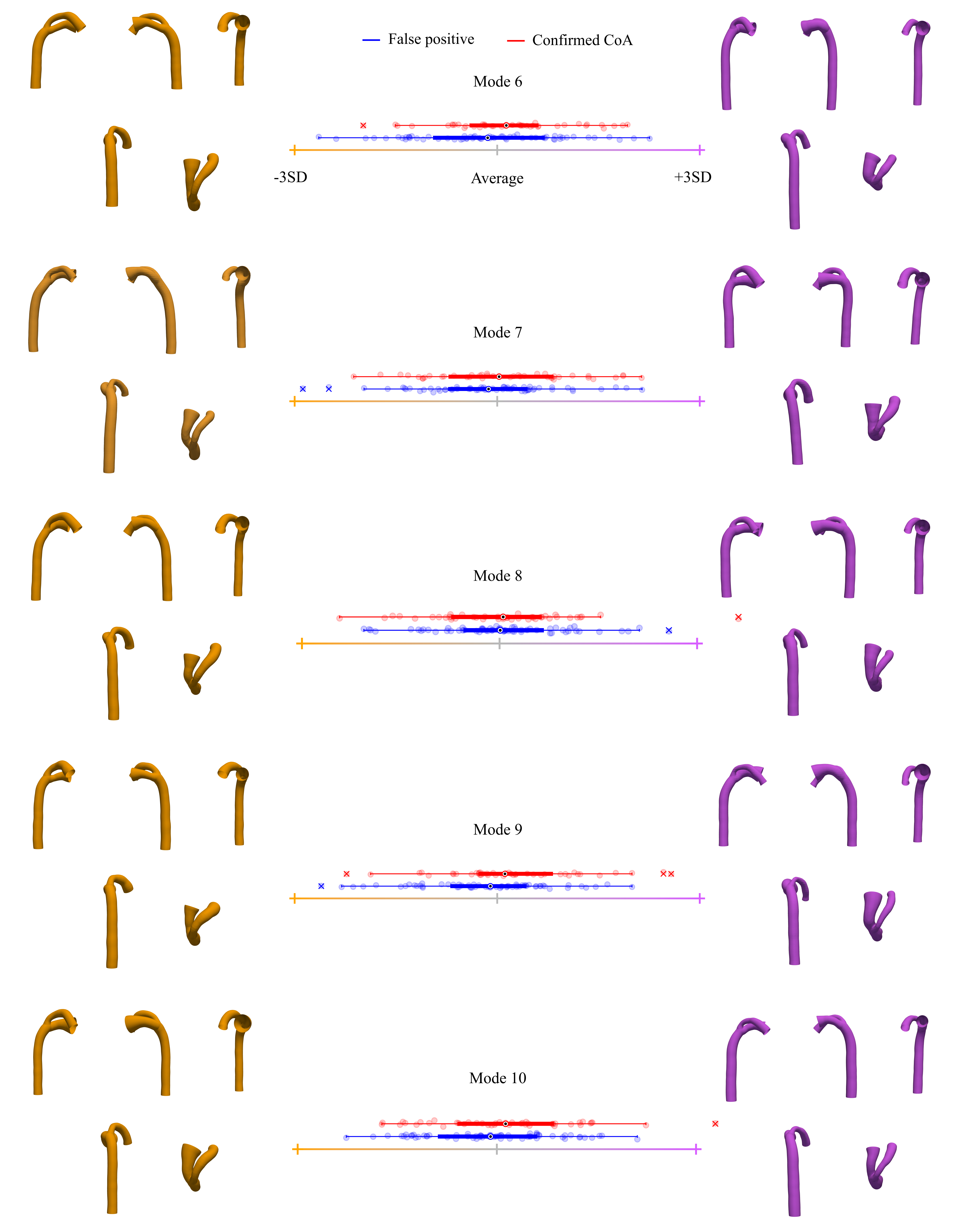

Supplement: Supplementary file 2 — (PNG 1967 KB) [file 12265_2022_10335_MOESM2_ESM.png]

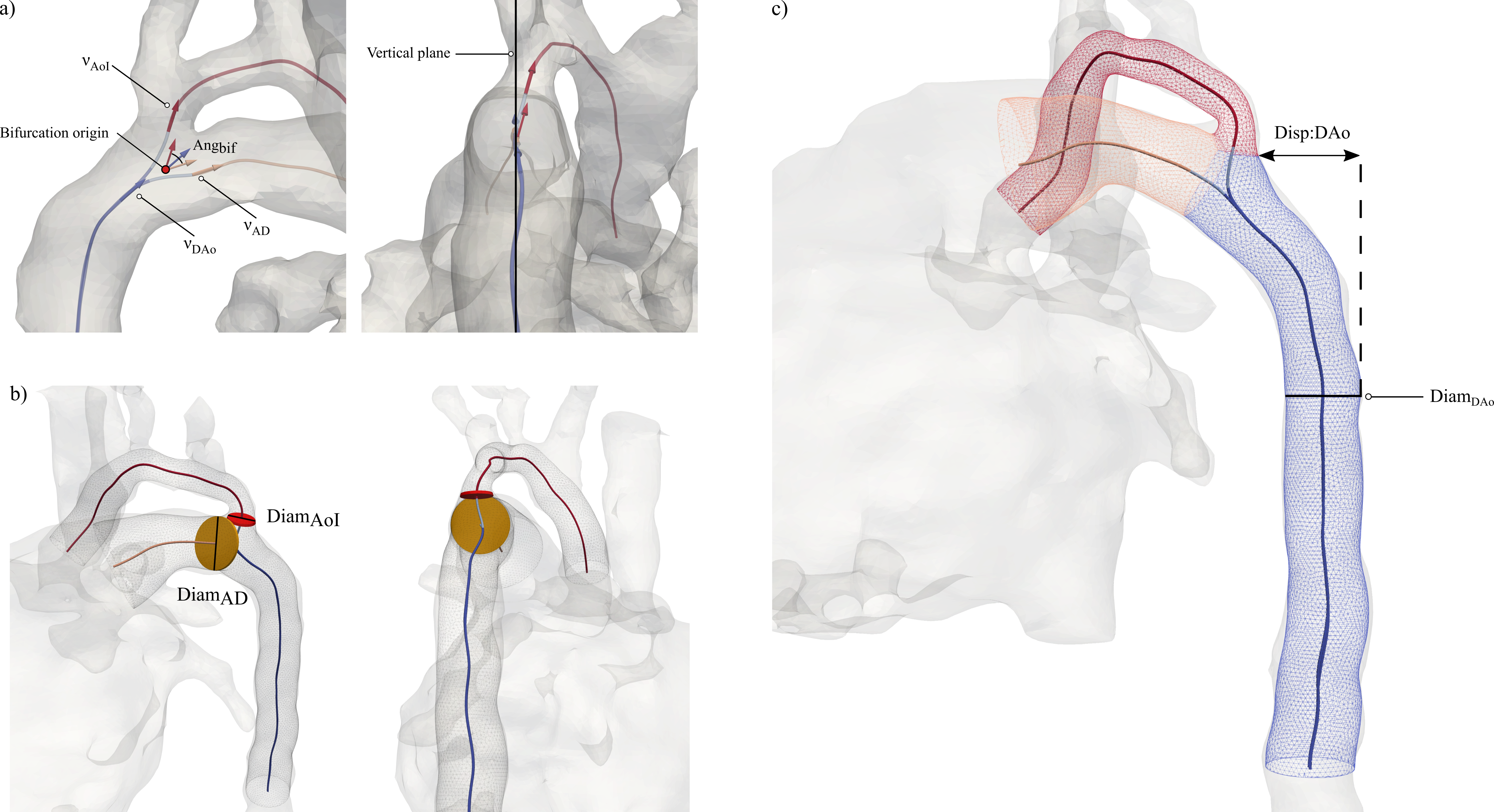

Supplement: Supplementary file 3 — (PNG 7253 KB) [file 12265_2022_10335_MOESM3_ESM.png]

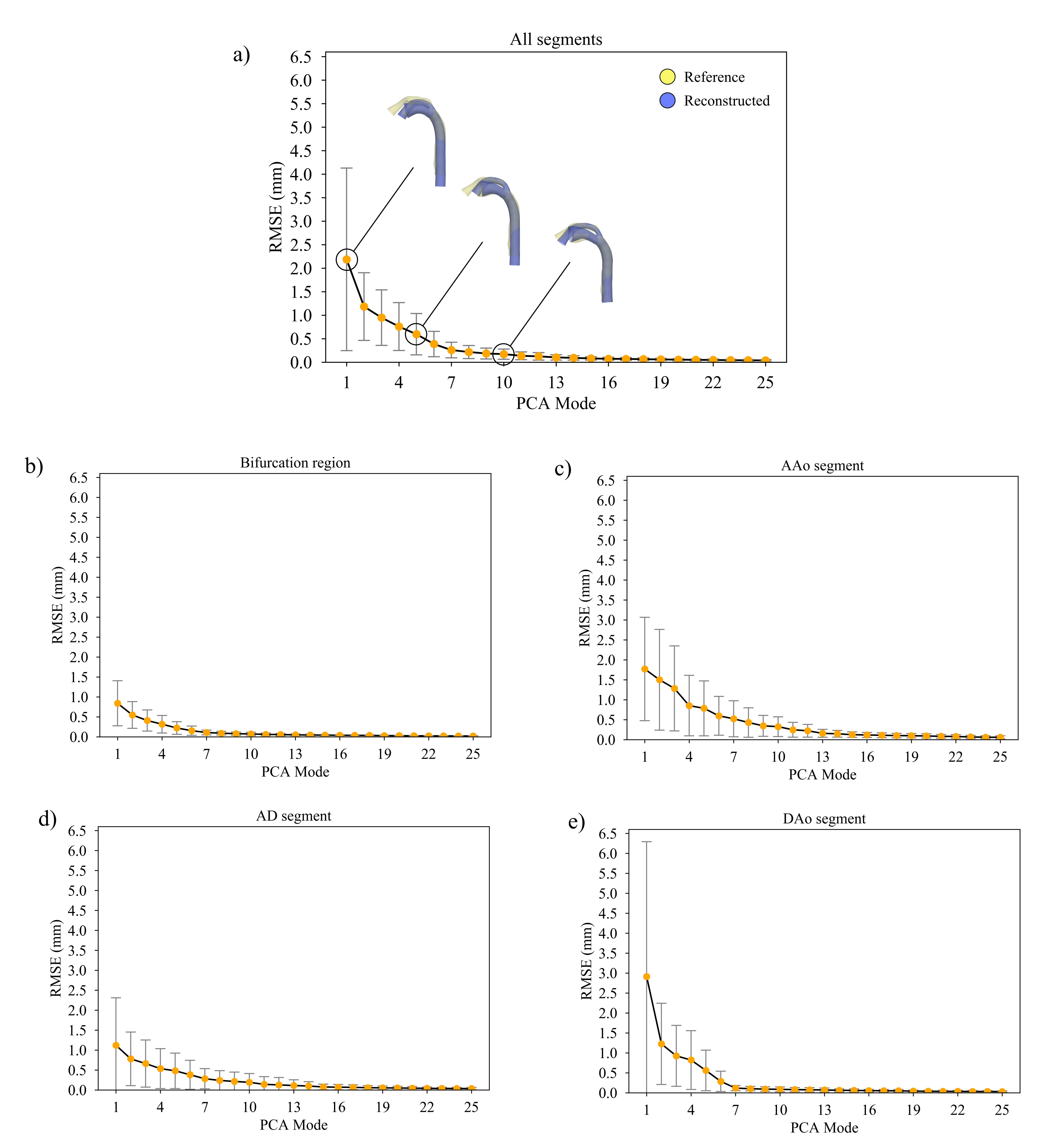

Supplement: Supplementary file 4 — (PNG 1078 KB) [file 12265_2022_10335_MOESM4_ESM.png]
