# Supplementary material for: Learning the Hidden Signature of Fetal Arch Anatomy: a Three-Dimensional Shape Analysis in Suspected Coarctation of the Aorta
Source: J Cardiovasc Transl Res. 2022 Oct 27;16(3):738–47. doi: 10.1007/s12265-022-10335-9 (PMC10299929; doi:10.1007/s12265-022-10335-9)
Supplement: Supplementary file 12 — (PDF 13.1 MB) [file 12265_2022_10335_MOESM12_ESM.pdf]

Supplementary Material - Learning the Hidden Signature of Fetal Arch Anatomy: A Three-dimensional Shape Analysis in Suspected Coarctation of the Aorta

Uxio Hermida, Milou P.M. van Poppel, David F.A. Lloyd, Johannes K. Steinweg, Trisha V. Vigneswaran, John M. Simpson, Reza Razavi, Adelaide De Vecchi, Kuberan Pushparajah, Pablo Lamata*

*Corresponding author: pablo.lamata@kcl.ac.uk

Building the statistical shape model

After extraction and alignment of centerline tracts and their associated maximal inscribed radius r, information of shape is encoded by a common vector space $R^{N(3+1)}$, where N is the number of centerline vertices of the three segments of interest: ascending aorta (AAo) until the aortic isthmus (AoI), arterial duct (AD) and descending aorta (DAo). A total of N=101 points were used to construct a single feature vector of length 4N for each case i:

$$x_i = [x_1, y_1, z_1, r_1, x_2, y_2, z_2, r_2, ..., x_N, y_N, z_N, r_N]^T \tag{1}$$

A Principal Component Analysis (PCA) can then be used to reduce the dimensionality of the 404 degrees of freedom and build the statistical shape model. PCA finds the directions that maximize the variance in the observed shapes around the population average, capturing the most common changes in a cohort (i.e., the linear directions of anatomical change from the population average, commonly referred as anatomical modes of variation or PCA modes). Due to differences in range of variation of radial features compared to coordinates, and to avoid biasing the influence of each feature in the resulting SSM [1], all features were standardized following:

$$x'_i = \frac{(x_i - \mu)}{\sigma} \tag{2}$$

where μ and σ are the per-feature mean and standard deviation (SD) vectors respectively. Each shape is therefore described by the vector x'_i and the average shape of the population can be computed as:

$$\bar{x} = \frac{1}{N_s} \sum_{i=1}^{N_s} x'_i \tag{3}$$

where N_s is the total number of samples in the cohort. Using $\bar{x}$ and x'_i, the covariance matrix Σ was computed by:

$$\Sigma = \frac{1}{N_s - 1} \sum_{i=1}^{N_s} (x'_i - \bar{x})(x'_i - \bar{x})^T \tag{4}$$

The anatomical modes of variation ϕ_m (eigenvectors or PCA modes) and their respective variances λ_m (eigenvalues) can be obtained by singular value decomposition (SVD) of Σ. Anatomical modes of variation are ranked by the amount of variance explained, so that the first modes represent the most robust shape features to acquisition and segmentation noise. As a result, the parametric space is reduced and each shape can be described by a small set of shape coefficients that define the linear combination of the first k PCA modes of variation:

$$\hat{x}'_i = \bar{x} + \sum_{m=1}^{k} b_m \phi_m \tag{5}$$

where b_m are the case-specific shape coefficients along each anatomical mode of variation ϕ_m, computed as:

$$b_m = \phi_m^T \cdot (x'_i - \bar{x}) \tag{6}$$

A two-tailed Student's t-test was used to determine differences between shape coefficients b_m for each subgroup in the population (CoA/false positives). P-values < 0.01 were considered as statistically significant. Extreme shapes were obtained for each linear axis of anatomical variation by considering b_m as $\pm 3\sqrt{\lambda_m}$ in (8) (i.e., $\pm$3SD).

3D visualizations of the shapes captured by the centerlines were obtained by first inverting the standardization scaling in (2) to bring the shape features into the original parametric space:

$$\hat{x}_i = \mu + \hat{x}'_i \sigma \tag{7}$$

Then, using $\hat{x}_i$, an implicit tube function was sampled on a 128 x 128 x 128 pixel grid to create an image that crosses zero at the boundary of the surface captured by the centerline given its radius r. A marching cubes algorithm was then used to obtain a 3D surface representation of $\hat{x}_i$. Finally, for the sake of visualization, endpoints of the resulting shape were clipped by Boolean subtraction with a tubular surface generated with information about centerline normal and radius at endpoints.

Shape changes in antenatally suspected coarctation of the aorta

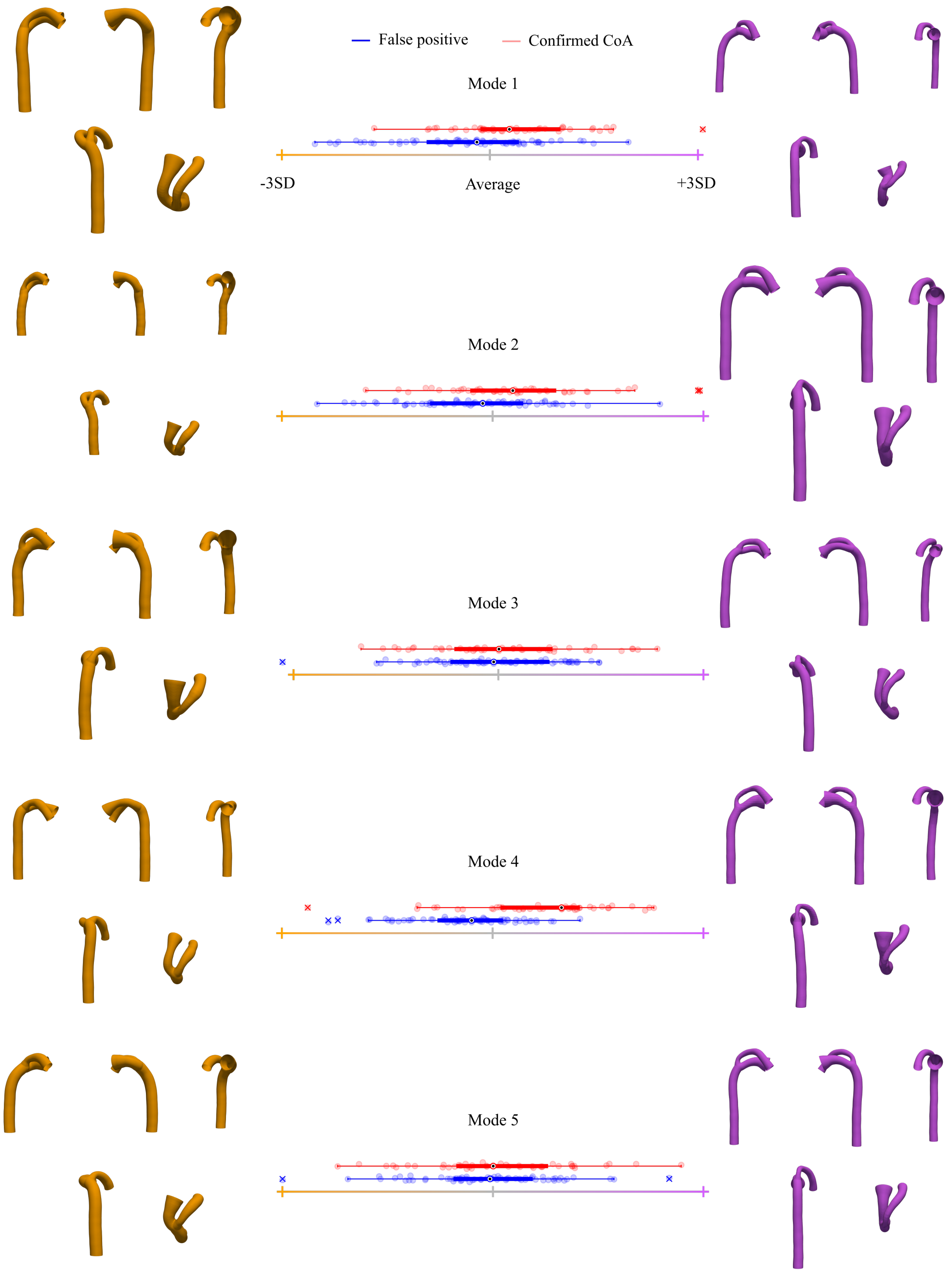

Figure S1. First 5 PCA anatomical modes of variation, showing differences between false positive (FP) and coarctation of the aorta (CoA) cases. Box-plots represent the FP (blue) and CoA (red) distributions of PCA scores along each axis. Orange cross and shapes show -3SD from the average shape; purple cross and shapes, +3SD. The average of the population is represented by a gray cross (PCA score 0). Modes 1, 2 and 4 showed significant differences between the two subgroups in the population (p-value <0.01). Those relevant modes show that FP cases tend to have a more aneurysmal arterial duct (AD) inserting laterally into the descending aorta (DAo) and aortic arch. Conversely, the CoA cases tend to have a more proximal insertion of the isthmus into the superior aspect of a non-aneurysmal AD. Global size changes, together with lengthening changes, were mostly captured by the first 2 modes of variation. All modes captured changes in the cross-sectional diameter across different segments, showing that CoA cases tend to have a smaller isthmus to AD ratio and a smaller AAo. A proximal isthmal displacement was also observed (see modes 1, 2, 3 and 4).

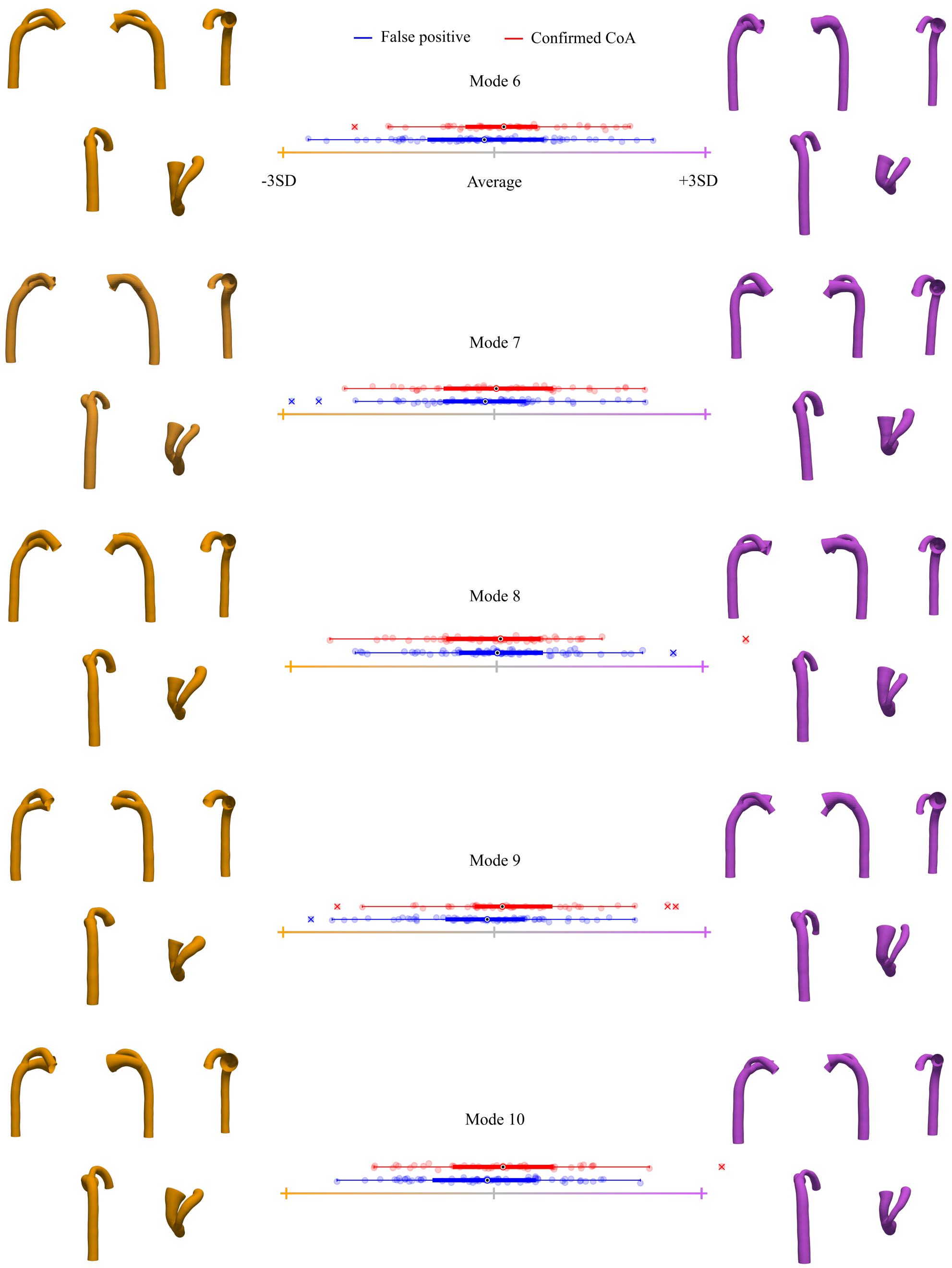

Figure S2. Modes of variation (5 to 10), showing differences between false positive (FP) and coarctation of the aorta (CoA) cases. Box-plots represent the FP (blue) and CoA (red) distributions of PCA scores along each axis. Orange cross and shapes show -3SD from the average shape; purple cross and shapes, +3SD. The average of the population is represented by a gray cross (PCA score 0). Most modes captured changes in cross-sectional diameter across different segments, as well as relative length changes. Variations in the angle between the different branches were also captured.

Baseline prediction with simple geometrical descriptors

All geometrical metrics were automatically extracted using the patient-specific centerlines and their captured surface. A set of 11 geometrical descriptors were studied. For a visual representation, see Figure S3.

- Bifurcation angle between AD and aortic isthmus (Ang_{bif}).
- Projected vertical angle between AD and aortic isthmus (Ang_{vert}).
- Ratio of the cross-sectional diameter of the aortic isthmus at bifurcation to that of the AD at bifurcation. (Isthmus:Duct ratio).
- Distance between the posterior wall of the isthmus and the posterior wall of the DAo (isthmal displacement), indexed to the cross-sectional diameter of the DAo (Disp:DAo).
- Cross-sectional diameter of: AD at bifurcation (Diam_{AD}), aortic isthmus at bifurcation (Diam_{AoI}), proximal AD (Diam_{pAD}), proximal AAo (Diam_{pAAo}), distal AD (Diam_{dAD}), distal AAo (Diam_{dAAo}). Proximal and distal diameters were computed as the mean value over the length of each half of the AAo/AD respectively.
- Vertical displacement of the transverse aortic arch with respect to the medial AD (Disp:Vert).

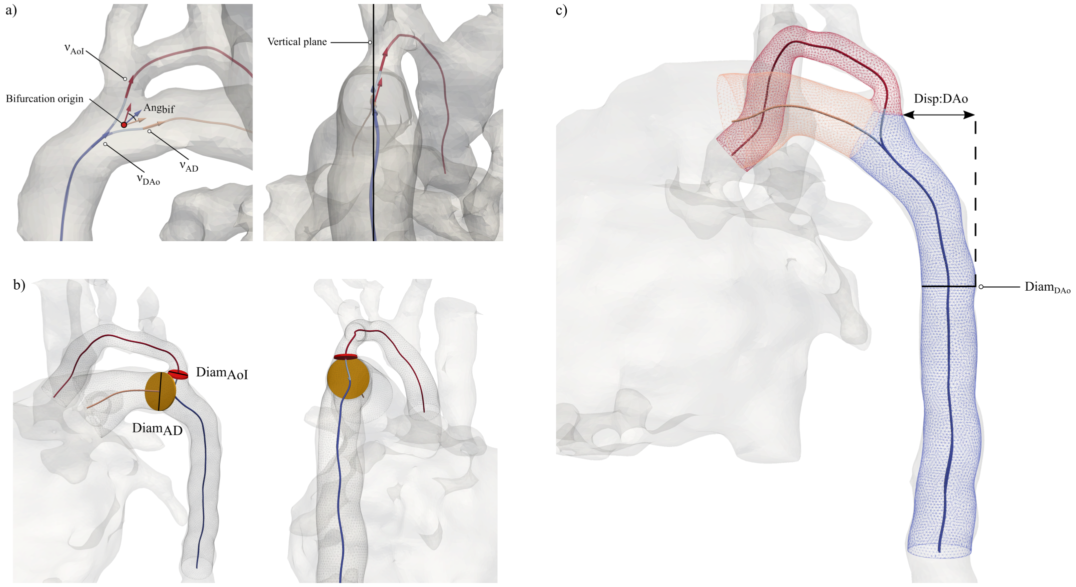

Figure S3. Schematic representation of the objective extraction of simple geometrical descriptors from three-dimensional fetal anatomies. All metrics are extracted using the patient-specific centerlines and their captured surface (showed as a wireframe). a) After geometrical decomposition of the arterial bifurcation and the centerlines into its constituent segments, a bifurcation vector for each segment is computed. The left panel shows the bifurcation vectors both at the start of each segment and reference bifurcation origin. The angle between the aortic isthmus vector (v_{AoI}) and the arterial duct (AD) vector (v_{AD}), Ang_{Bif}, is depicted. The right panel shows the vertical plane used to project each bifurcation vector and used to obtain the projected vertical angle between the AD and aortic isthmus (Ang_{vert}); b) Visual representation of the cross-sectional diameter used to compute the ratio between the aortic isthmus (AoI) and AD. The cross sectional diameter is computed at the starting branch point (as computed after decomposition of centerlines). c) The distance between the posterior wall of the aortic isthmus and the posterior wall of the DAo, indexed by the cross-diameter of the DAo ($Diam_{DAo}$), is shown (Disp:DAo).

Table S1 shows the discriminative performance of each simple geometrical descriptor, as well as their R2 with respect to the CoA shape risk score.

Table S1. Discriminatory Performance of Each Anatomical Descriptor

Score	AUC	R^2
Ang_{bif}	0.727	0.15
Ang_{vert}	0.809	0.47
Isthmus:Duct ratio	0.711	0.20
Disp:DAo	0.716	0.21
Diam_{AD}	0.708	0.18
Diam_{AoI}	0.694	0.20
Diam_{pAD}	0.711	0.19
Diam_{pAAo}	0.588	0.06
Diam_{dAD}	0.673	0.12
Diam_{dAAo}	0.762	0.33
Disp:Vert	0.724	0.26
CoA shape risk score	0.907	

Ang_{bif}: Bifurcation angle between arterial duct (AD) and aortic isthmus; Ang_{vert}: Projected vertical angle between AD and aortic isthmus; Isthmus:Duct ratio: ratio of the mean cross-sectional diameter of the aortic isthmus to that of the AD; Disp:DAo: Distance between the posterior wall of the aortic isthmus and the posterior wall of the DAo (isthmal displacement), indexed to the size of the DAo; Diam_{AD}: Cross-sectional diameter of the AD at insertion in bifurcation; Diam_{AoI}: Cross-sectional diameter of the aortic isthmus (AoI) at insertion in bifurcation; Diam_{pAD}: Mean cross-sectional diameter of the proximal AD; Diam_{pAAo}: Mean cross-sectional diameter of the distal AD; Diam_{dAD}: Mean cross-sectional diameter of the distal AD; Diam_{dAAo}: Mean cross-sectional diameter of the distal AAo; Disp:Vert: Vertical displacement of the transverse aortic arch with respect to the medial AD.

Iterative reconstruction of cases with PCA modes

As a result of the dimensionality reduction of PCA, each anatomy $\hat{x}'_i$ can be reconstructed by sequentially adding the information contained in each subsequent PCA mode k to the average shape $\bar{x}$:

$$\hat{x}'_i = \bar{x} + \sum_{m=1}^{k} b_m \phi_m \tag{8}$$

where b_m are the case-specific shape coefficients along each anatomical mode of variation ϕ_m, computed as:

$$b_m = \phi_m^T \cdot (x'_i - \bar{x}) \tag{9}$$

Therefore, we explored the reconstruction error (i.e., root mean squared error (RMSE)) between each reconstructed shape $\hat{x}'_i$ depending on the k PCA modes included and the reference shape x_i for each case i. RMSE was explored for each centerline segment separately (i.e., AAo, AD, DAo), for all three grouped segments, and the

centerline points closer to the isthmal insertion (i.e., half of the points for each centerline segment).

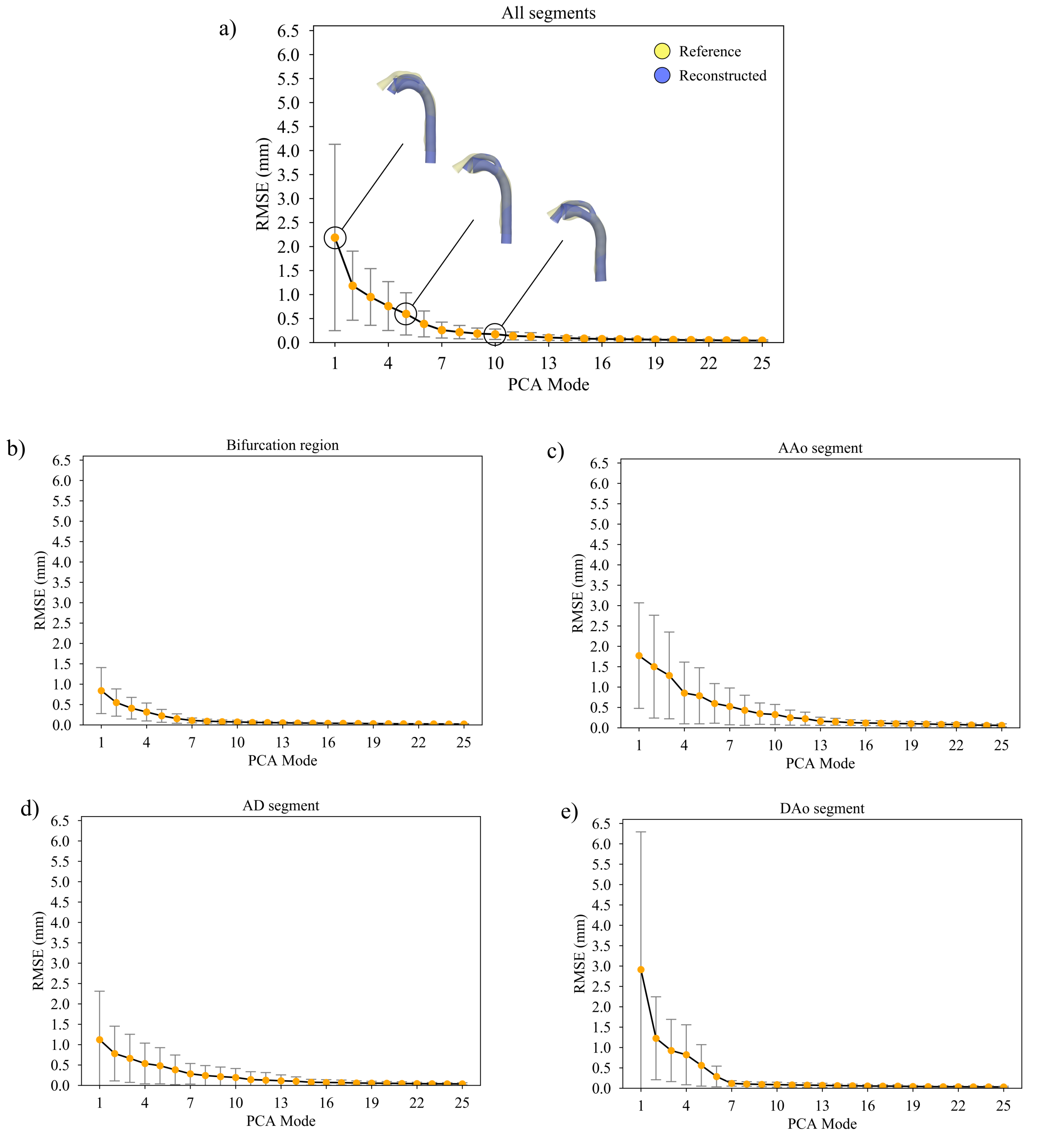

Figure S4. Error bar plots showing the reconstruction error (root mean squared error (RMSE)) depending on the number of PCA mdoes for a) All centerline segments; b) Centerline points close to the bifurcation (i.e., isthmal insertion point); c) Ascending aorta (AAo) segment; d) Arterial duct (AD) segment; e) Descending aorta (DAo) segment.

References

[1] Gewers, F. L. et al. (2021). "Principal component analysis: A natural approach to data exploration". *ACM Computing Surveys* 54.4, pp. 1–33. DOI: 10.1145/3447755.
